# A Comparative Analysis of Carbon Footprint in the Andalusian Autochthonous Dairy Goat Production Systems

**DOI:** 10.3390/ani13182864

**Published:** 2023-09-09

**Authors:** Juan Manuel Mancilla-Leytón, Eduardo Morales-Jerrett, Sara Muñoz-Vallés, Yolanda Mena

**Affiliations:** 1 Departamento de Biología Vegetal y Ecología, Facultad de Biología, Universidad de Sevilla, 41012 Sevilla, Spain; 2 Departamento de Agronomía, Escuela Técnica Superior de Ingeniería Agronómica, Universidad de Sevilla, 41013 Sevilla, Spain; jerrett@us.es (E.M.-J.); saramval@us.es (S.M.-V.); yomena@us.es (Y.M.)

**Keywords:** carbon sink, feed management, standardization equations, environmental sustainability

## Abstract

**Simple Summary:**

The carbon footprint (CF) is, at present, the most widely used indicator to quantify the impact of livestock farming on global warming, fulfilling also the purpose of identifying production practices that develop more efficient uses of available resources, as well as ways to minimize their environmental impact. The aim of this study was to characterize the CF in the four different production systems of autochthonous dairy goat breeds currently occurring in Andalusia (S Spain), from confined to pastoral systems, also considering the carbon sink ability by vegetation associated with land-based livestock systems. Despite the difficulties of calculation, the relevance of using a species-specific standardization equation and of taking into account land carbon sink ability was demonstrated. The four production systems analyzed obtained similar CF values, all showing room for improvement. This must be translated into the adoption of specific actions for each production system and territory, particularly regarding the improvement of grazing activity, optimal use of farm resources, and appropriate management of manure or the use of local food. Professional advice, training, and the use of specific management tools are essential for the implementation of these strategies to move towards low-carbon goat production.

**Abstract:**

The small ruminant livestock sector faces the challenge of reducing greenhouse gas (GHG) emissions. Carbon footprint (CF) studies on dairy goats, the most widely used indicator to quantify the impact of livestock farming on global warming, are still few. The aim of this study was to calculate the CF of the different production systems of autochthonous dairy goat breeds presently occurring in Andalusia (S Spain) and identify systems and practices that can minimize their environmental impact in these terms. Twenty-one farms were monitored during a year, obtaining valuable information that allowed the CF calculation on a “cradle-to-gate” approach, taking into account both GHG emissions at the farm level and carbon sink by vegetation associated with land-based systems. Results showed similar CF values for the analyzed systems (1.42, 1.04, 1.15, and 1.17 kg CO_2_-eq kg^−1^ fat–protein corrected milk for indoor systems without associated crops, indoor systems with associated crops, grazing systems with high feed supply, and pastoral systems, respectively). To minimize their environmental impact, specific actions must be developed for each system, particularly regarding genetic improvement, reproductive and feeding management, including pasture management, and the integration of livestock activity into the bio-circular economy with the help of professional advice.

## 1. Introduction

Climate change is a real fact affecting the entire planet through dangerous effects such as rising average temperatures and sea levels, melting ice in the Arctic, or an increase in extreme events such as heat waves, floods, or droughts. This climate change is promoted by the increasing phenomenon of global warming, which is motivated by the emission of so-called “greenhouse gases” (GHG) as a result of human activities, which have the ability to increase the capacity of the Earth’s atmosphere to retain heat [1].

Since 2014, climate change has been one of the nine specific objectives against which the European Commission assesses the performance of the Common Agricultural Policy. The current EU policy framework (2030 Climate Target Plan) aims to reduce GHG emissions by 40% by 2030 and by 55% by 2050, thus achieving net zero emissions by that date [2]. In this context, it is recognized that some of the current livestock production models contribute to the emission of the main greenhouse gases responsible for global warming (sheep and goats are responsible for about 6.5% of the global livestock sector’s emissions [3]). In recent years, the livestock sector has been faced with the difficult challenge of reducing GHG emissions while, at the same time, responding to the significant increase in demand for livestock products, mainly driven by the growth of the world’s population, improved economic well-being, urbanization, poor dietary habits, and current marketing models, among other factors [4]. The production and processing of food, together with emissions during enteric fermentation in the case of ruminants, are the two main sources of GHG emissions linked to the livestock sector, being, respectively, responsible for 45% and 39% of the total emissions from the activity. Additionally, manure storage and processing represents 10%, the remaining part being attributed to the processing and transportation of livestock products [5]. On the other hand, the livestock sector also has to deal with a number of other issues, including economic viability and the threat posed by the growing social perception that livestock farming, in general, and ruminant farming, specifically, is a major contributor to the phenomenon of climate change.

In this background, the most prominent method for quantifying the total GHG emissions (expressed as carbon dioxide equivalent; CO_2_-eq) associated with a product is the carbon footprint (CF). The process of calculating the CF additionally allows the evaluation of the main emission processes and the identification of needs for the implementation of mitigation strategies. However, there are major methodological difficulties in calculating the CF for a small ruminant farm due to the lack of information on its management and the complexity of such systems in terms of extension and boundaries, use of the appropriate functional unit, type of allocation, etc. [6,7].

To date, CF studies on small ruminants, particularly dairy goats, are still few and far between in terms of approach, methodology, and results interpretation, even more so in their consideration of natural carbon sinks in the CF calculation. These studies often involve production systems that are not always representative of the sector, or the results obtained cannot be compared between studies because of the methodology used (e.g., different standardization of milk) or due to a lack of information (identifying the scope of the system, allocating GHG emissions to by-products, etc.) [7]. As a result, GHG emission intensity (expressed as emissions per unit of animal product) can vary considerably between production units, even in similar production systems. To all this must be added the difficulty of taking into account the carbon sink ability of the soil and vegetation during the CF calculation, in particular in those farms that have a territorial basis for the use of livestock [8,9].

Southern Europe, and more specifically Andalusia (southern Spain), is particularly threatened by climate change (a general increase in temperature with maximum values of +6.5 °C and a 20–30% reduction in annual rainfall are predicted) [10], where agricultural productivity would be significantly severely compromised. In this sense, livestock systems (mainly ruminants) able to adapt and use endogenous resources, for example, through grazing, can provide an opportunity for sustainable food production in such arid contexts [11]. Recent studies have also shown that the production of wheat crops dedicated to low-yielding animals such as buffaloes significantly affects the category of Agricultural Land Occupation [12]. On the other hand, soil health and fertility and carbon sink ability by both soil and vegetation can be favored by properly managed ruminant grazing. In this sense, pasture-based carbon sequestration can mitigate, to varying degrees, the GHG emitted by the livestock it feeds as well [13,14]. Previous studies have shown that taking carbon sinks into account in the net GHG emission balance significantly reduces emissions per unit of product on farms with land-based grazing [6,8,15].

Andalusia is the most important goat milk producer region in Spain, with around one million goats [16]. Contrary to the situation in other livestock sectors, the evaluation of goat production in Andalusia has undergone profound changes since the 1980s, and the development of this sector has led to great heterogeneity in terms of the breeds used, the area in which the activity is carried out, and the different management systems that have been established (from the stabled system to the grazing system, in which more than 70% of the feed is taken from grassland and shrubland) [17]. These different management systems should enable the sector to implement different strategies to adapt to the new climatic and social framework, reduce its CF, and maintain its current image of sustainability [18]. It is important to know how these systems behave in relation to GHG emissions and thus be able to promote them while reducing their impact, as well as to propose emission reduction strategies in each case.

The objective of this study was to characterize the CF in different production systems of autochthonous dairy goat breeds currently occurring in Andalusia (S Spain), including the carbon sink ability by vegetation associated with land-based livestock systems to feed their animals. It is expected that the CF values of pastoral dairy systems will be reduced by using fewer outputs (concentrate and forage supply, electricity, fertilizer, etc.) and by incorporating the sink capacity of vegetation into calculations. This will help to identify systems and practices that develop a better use of available resources, as well as ways to minimize their environmental impact.

## 2. Materials and Methods

### 2.1. Study Area and Farms Selection

The region of Andalusia (S Iberian Peninsula) extends over 87,600 km. It includes three major mountain ranges: Sierra Morena and the Baetic system, consisting of the Subbaetic and Penibaetic mountains, separated by the Intrabaetic Basin. Lower Andalusia is in the Baetic Depression of the valley of the Guadalquivir River [19]. This region, with a wide variety of climatic zones (coastal, inland, mid-mountains, high mountains, and semi-arid and arid climates), is strongly influenced by subtropical anticyclones, with marked differences during mild-humid winters and very dry summers [20,21]. From a demographic point of view, Andalusia has 8.47 million inhabitants, where 17.5% of the total population resides in rural areas, which occupy 65.0% of the inhabited Andalusian surface.

A sample of twenty-one farms belonging to the Federación Andaluza de Asociaciones de Ganado Caprino de Raza Pura (Cabrandalucia) were monitored monthly throughout 2018 to collect key information about inputs and outputs and animal management practices in order to calculate their CF according to Gutierrez-Peña et al. [6]. These farms were representative of the dairy goat systems described by Morales-Jerrett et al. [17] for the autochthonous dairy goat breeds present in Andalusia: (i) indoor systems without associated crops (IS), (ii) indoor systems with associated crops (ISC), (iii) grazing systems with high feed supply (GS), and (iv) pastoral systems (PS). Four autochthonous goat breeds were included in the study: Florida, Malagueña, Murciano-Granadina, and Payoya.

Besides a suitable methodology, due to the diversity of production systems, a clear classification of the different dairy goat production systems is necessary for proper analysis and decision making to minimize their environmental impact. The classification made by Morales-Jerrett et al. [17], based on the use of autochthonous breeds in Andalusia, placed, at one extreme, goat farms with permanent stabling and outdoor exercise yards (IS group). These farms are characterized by non-seasonal productivity, which responds to the demands of the dairy industry, and a high dependence on feed from outside the farm, subject to the volatility of international markets for raw materials for animal feed. At the other extreme, there are goat farms adapted to grazing, usually multispecies, which use different Mediterranean grasslands and shrublands according to their spatial and temporal availability (PS group). Two intermediate models (GS and ISC groups) complete the range of possibilities between these two systems. Technical and economic characteristics, based on the calculation of indicators, of each production system are shown in the referred study [17].

### 2.2. Data Collection and Indicators Calculation

Data collected for each study farm were recorded using the AMALTEUS 1.0 management software, specifically developed for goat farming by the University of Seville and Cabrandalucia, and using cloud storage technology [22]. The use of this type of digital tool represents an opportunity to generate data periodically on a larger scale with a single, standardized methodology. In AMALTEUS, data collection is carried out in different stages, depending on their nature. First, a registration form and a social questionnaire are filled in with the help of a technician. Further, farmers have to make periodic entries as events occur concerning census, births and deaths, feed purchased, income, and expenses by using the app installed on their mobile devices. By the end of the year, data related to amortizations, feedstocks, crops and pastures, labor, and subsidies are also requested to be introduced. All data were recorded on the web and were accessed only by authorized personnel, thus conferring maximum security and privacy in relation to data protection.

### 2.3. Carbon Footprint Calculation

The CF was calculated on a “cradle-to-gate” approach, taking into account all GHG emissions at the farm level: (i) those due to direct animal inputs (enteric fermentation and manure management); (ii) those derived from soil management; and (iii) those caused to inputs (feed, fertilizer, energy, etc.). Machinery, buildings, medicines, and other minor inputs were excluded from the assessment [6].

For the calculation of GHG emissions (CH_4_, N_2_O, and CO_2_) in the study farms, the current PAS 2050 standard and the guidelines established in the IPCC Guidelines were followed [23]. Emissions were expressed in CO_2_ equivalents (CO_2_-eq) according to the IPCC 100-year global warming potential (CO_2_ =1; CH_4_ = 28; N_2_O = 265). The GHG emissions from animal enteric fermentation were performed at Tier 1 according to the updated IPCC Guidelines [23]. The emission factors used were obtained mainly from the Ecoinvent database, as well as from different public sources (e.g., the European Environment Agency and the Spanish Ministry for Ecological Transition and Demographic Challenge).

Emissions were expressed as CO_2_-eq per kg of fat and protein-corrected milk (FPCM), as recommended by the most common Life Cycle Analysis guidelines for the dairy sector [24]. The obtained results were expressed according to 3 different specific standardization equations for goat’s milk: (1) the equation proposed by Mancilla-Leytón et al. [7] specifically developed for dairy goat’s milk with a fat content above 4%, FPCM (kg) = raw milk (kg) × [(0.10 × fat content (%) + 0.08 × protein content (%) + 0.20)]; (2) the equation proposed by Robertson et al. [25] from the equation developed by Clark et al. [26] for dairy cow’s milk, FPCM (kg) = raw milk (kg) × [0.145 × fat content (%) + 0.092 × protein content (%) + 0.3]; and (3) the equation proposed by FAO [24] specifically developed for small ruminants, FPCM (kg) = raw milk (kg) × [0.1226 × fat content (%) + 0.0722 × protein content (%) + 0.0621 × lactose content (%)]. Since lactose data were not available, the default value of 4.8% was used as indicated in the guideline.

In parallel to the GHG emissions, carbon sink by vegetation was calculated for the farming systems with associated territories for feeding (ISC, GS, and PS), according to the methodology proposed by Muñoz-Vallés et al. [8]. SIOSE-based C sink was used to obtain the values of the natural vegetation of each ecosystem used by the goats. Regarding crops, a maximum standard sink value of 0.1 kg of CO_2_ per m^2^ and year was applied [8].

Finally, the CF (considered as emissions minus sinks) was expressed in kg of CO_2_-eq kg^−1^ FPCM. The allocation principle applied was economic [6]; its calculation was based on the income from the sale of milk (90–94%) and the sale of kids (6–10%) obtained for each farming system under study.

### 2.4. Statistical Analysis

ANOVA and Tukey test were used to test for possible significant differences between study groups. Prior to this, normality and homoscedasticity tests were assessed. IBM SPSS Statistic 23.0 for Windows (SPSS Inc., Chicago, IL, USA) was used for all analyses.

## 3. Results and Discussion

### 3.1. Relevance of the Standardization Equations Used to Calculate kg of FPCM

Depending on the specific standardization equations used for calculating kg of FPCM for goat milk production, the CF can vary significantly [7]. Pardo et al. [27] compare the results of CFs among different dairy small ruminant productive systems, revealing the great difficulty of making such a comparison due to the heterogeneity of methodologies applied. Most studies carried out on goat milk production do not even detail the calculation procedure or use the specific calculations developed by Pulina et al. [28] for sheep or the modification of Robertson et al. [25] by Clark et al. [26] for dairy cows. Only a few studies, such as Pardo et al. [27], use the standardization equation suggested by FAO [24] for small ruminants where the milk yield was corrected at 4.0% fat and 3.3% protein to provide a comparison with dairy cow milk. This formula requires knowledge of the lactose content of the milk, which is not usually available in the information provided by the industry to the farmer, as it is not a payment criterion. Likewise, this parameter is not available in the official background information on milk control provided by the competent state ministry [29]. Therefore, the use of this standardization equation is limited unless the lactose content (4.8%) is replaced by a fixed value as specified in the IPCC Guidelines [24].

**Figure 1:**
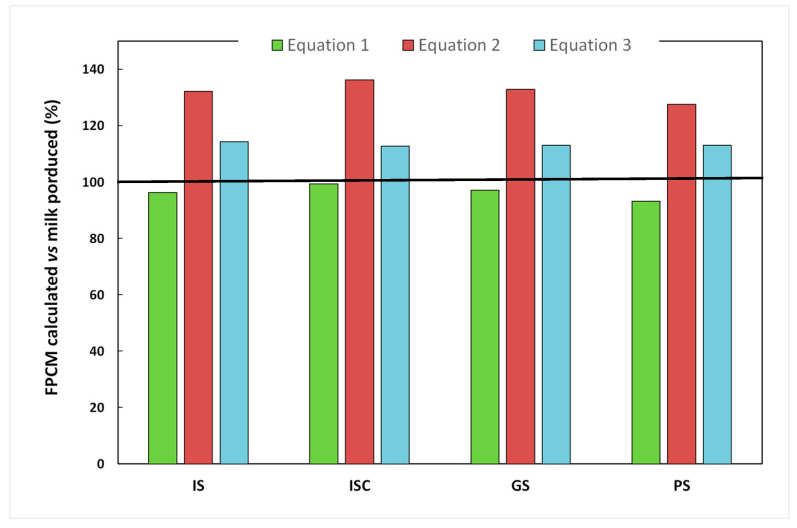
Comparison of the real kg of milk production data with respect to the values obtained (kg of fat and protein corrected milk, FPCM) from each of the standardization equations for goat milk used. The real value of milk production is represented as 100% (black line). Equation (1) proposed by Mancilla-Leytón et al. [7]; Equation (2) proposed by Robertson et al. [25]; and Equation (3) proposed by FAO [24]. IS: Indoor systems without associated crops (*n* = 8); ISC: indoor systems with associated crops (*n* = 3); GS: grazing systems with high feed supply (*n* = 5); and PS: pastoral systems (*n* = 5).

In the present study, considerable variations were found between the kg of FPCM values according to the specific standardization equation used (Figure 1). The kg of FPCM values obtained from Equation (2), proposed by Robertson et al. [25], increased between 28 and 34%, and from Equation (3), proposed by FAO [24], increased between 14 and 16% with respect to the real values of milk production. Gutierrez-Peña et al. [6] also found a 41% difference in CF values for different dairy goat systems, depending on the equation used. These values are similar to those obtained by Mancilla-Leytón et al. [7], who, using FPCM calculations for sheep, found an overestimation of 35% due to the higher fat content of sheep milk compared to goat milk. In the case of Equation (1), proposed by Mancilla-Leytón et al. [7], the obtained kg of FPCM values obtained decreased between 2 and 7% with respect to the real values of milk production (Figure 1). These results indicate that the use of a specific equation for goat’s milk is much more appropriate for the calculation of their CF.

### 3.2. Diversity of Goat Production Systems and CF of a kg of Milk

In terms of inputs, significant differences were found only for feed. The values showed that the concentrate supply provided to each goat was significantly lower in the PS system (almost 50% less) than in the rest of the farming system (*p* = 0.001). For the rest of the system, the values were similar (465–568 kg goat^−1^ year^−1^; *p* ≥ 0.05). In terms of outputs, the PS system presented significantly lower kg of milk sold per goat values (35–45% less, *p* = 0.013) than the rest of the farming systems, while the rest of the systems presented similar values (489–578 kg goat^−1^ year^−1^; *p* ≥ 0.05). No significant differences were found among the farming systems studied for the rest of the variables analyzed (Table 1, *p* ≥ 0.05).

The calculation of GHG emissions, and therefore CF values, reflected these differences found in each of the identified systems. Although the values for total GHG emissions were slightly higher in systems where grazing is present (GS and PS systems), no significant differences were found (*p* ≥ 0.05) (Table 2). CF values for indoor systems in this study (IS and ISC), obtained from the FAO standardization equation [24], are similar to those reported by Pardo et al. [27] (0.93 kg CO_2_-eq kg^−1^ FPCM, based on results by Robertson et al. [25]) and were lower, for the same systems, than those found by Pardo et al. [27] (1.41–1.73 kg CO_2_-eq kg^−1^ FPCM). The CF values obtained in the present study for the grazing systems (GS and PS), based on the equation of Robertson et al. [25] and taking into account the carbon sink ability, are very similar to those obtained by Gutiérrez-Peña et al. [6] for the goat grazing systems located in the same study area (1.04–1.40 kg CO_2_-eq kg^−1^ FPCM).

**Figure 2:**
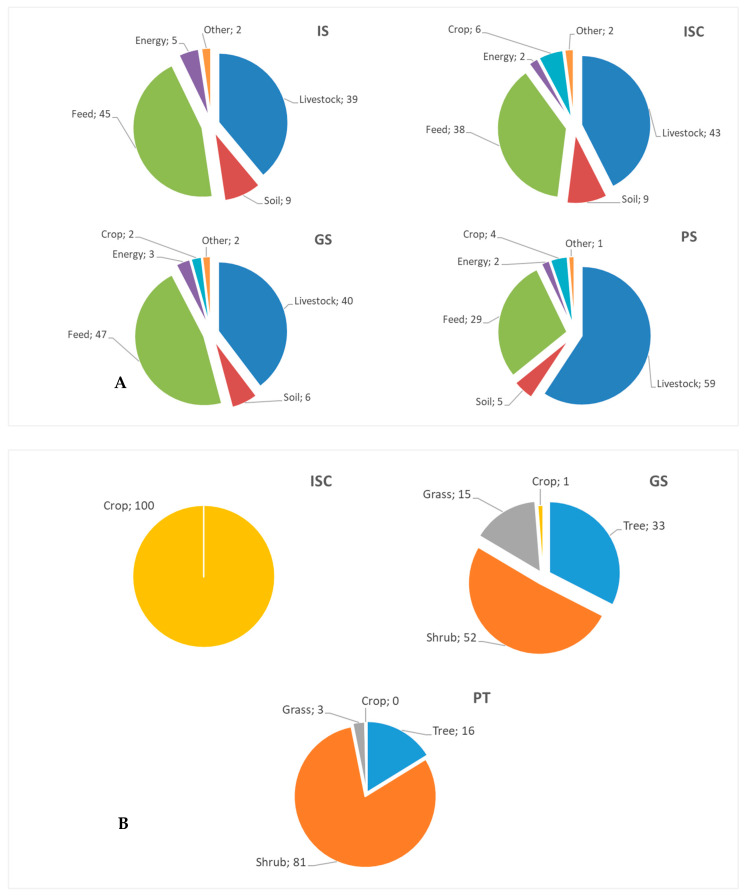
Contribution of the different pollutant sources of each goat farming system activity to the total greenhouse gas emissions (**A**). Contribution of different land uses of each goat farming system in carbon sequestration (**B**). IS: Indoor systems without associated crops (*n* = 8); ISC: indoor systems with associated crops (*n* = 3); GS: grazing systems with high feed supply (*n* = 5); and PS: pastoral systems (*n* = 5).

For all the production systems studied, the biological activity of the goats (mainly enteric fermentation) and the use of purchased feed were the main sources of emissions (Figure 2A). Depending on the system, livestock emissions could account for 39–60% of total emissions and purchased feed emissions for 30–47% of the total emissions. Soil emissions were the next main source of pollution, with values around 5–10% of total emissions. Finally, emissions from the use of energy (fuel and electricity) and crops (inorganic fertilization) were the minor sources of emissions with values equal to or less than 5% of total emissions (Figure 2A). In line with other small ruminant studies, enteric fermentation, together with feeding, was the source responsible for the highest percentage of emissions [6,30,31,32]. The same emission factor (Tier 1) was used for all systems studied for enteric fermentation in goats. Therefore, the contribution of enteric fermentation to total GHG emissions is determined by the level of grazing carried out in each of the systems; the greater the grazing, the lower the input required for feeding, and, therefore, the greater the contribution of livestock emissions to total emissions.

In practice, the difficulty and complexity of measuring carbon sinks related to livestock activities have led to their exclusion from most published CF studies, providing a biased view of reality [6,30,33,34]. In addition, current regulation does not provide enough guidance on how sinks need to be included in a methodological and standardized way. Including sink values in the calculation of the CF is an aspect that allows the real impact of farms on global GHG emissions to be better assessed by offsetting and reducing total emissions, often significantly [35]. The studied farms differed in their level of land use for pasturing, which was also inversely related to intensification level; carbon sink values found ranged between 1 and 2.9 t CO_2_ ha^−1^, which represented a considerable carbon compensation (Table 2). The values found in the PS system were significantly higher than in ISC and GS (86% and 70% higher, respectively, Table 2). The contribution of the different land uses in carbon sequestration was different according to the system studied: crops were responsible for 100% in the ISC; woodland + shrubland (>85%) in the GS; and mainly shrubland (>80%) in the PS (Figure 2B). The inclusion of carbon sink values had a significant effect on the CF values, so grazing systems (GS and PS groups) had lower values compared to intensive systems (12–32%, Table 2). Despite this reduction, the differences between the grazing systems were not significant due to the high variability between the farms that make up these systems (Table 2). Gutierrez-Peña et al. [6] also found differences between systems according to intensity, which were lower in the more pastoral systems.

### 3.3. Identification of Practices Which Contribute to the Environmental Sustainability of Dairy Goat Farming System Study

The CF is, at present, one of the most widely used indicators to quantify and reduce the impact of livestock on global warming [36]. Based on the obtained results, it can be stated that the use of the CF to assess farming must be subjected to certain considerations, particularly (i) the need to use the same milk standardization equation, being specific for each livestock species; (ii) preferably using the CF to analyze the evolution of each productive unit, during wide periods of time, with the aim of verifying the suitability of management strategies applied in terms of contribution to the reduction of farm GHG emissions; (iii) the need to promote the use of specific, reliable, and easy-to-use tools and software for farmers and technicians, allowing the assessment of CF evolution associated with changes in livestock management; and (iv) including carbon sink by vegetation in the CF of land-based systems for animal feeding, where appropriate.

Our results showed similar CF levels in the four analyzed groups, with room for improvement in each case. These models require management and operation strategies adapted to their differential characteristics in order to not only guarantee the profitability of the activity but also reduce its environmental impact. Such strategies cannot be generic and need to be adapted to the reality of each territory and productive model; nevertheless, they must consider, at least, the following elements: (i) genetic improvement as a means to obtain more efficient and adapted units to the territory; (ii) reproductive control as a basic element to minimize the unproductive periods of the goats; (iii) proper nutritional management, based on specialized advice, training of technicians and farmers, and optimization of the use of available resources; and (iv) the integration of livestock activity in the bio-economy of the territory, through the adequate management of manure and other by-products, or the preferential use of local resources, both in the manger and through rational use of grazing surfaces. Thus, the recommendations of the FAO for the substantial reduction of GHG emissions in livestock production are fulfilled: (i) an increase in productivity and food efficiency, (ii) carbon sink through grazing, and (iii) integration into the circular bio-economy [37].

Every analyzed model, but particularly the pastoral system (PS), needs to improve their milk productivity through genetic improvement, the elimination of unproductive animals, and the improvement of reproductive management, with the aim of shortening the periods in which the goats remain dry off [38]. This increase in productive efficiency and optimization of breeding goats would lead to a significant decrease in methane emissions resulting from enteric fermentation. The implementation of digitization in the goat sector (smart farming), as has already been carried out in other sectors through tools that contribute to precision farming, represents a great opportunity for farmers in this sector. In Andalusia, the autochthonous goat breeds associations comprising Cabrandalucia have been working for years on the comprehensive management of their farms, being pioneers in its digitization through the implementation of a modern enterprise resource planning (ERP) system and the development of specific computer tools, such as RUMIA [27] and AMALTEUS [22], the latter developed together with the University of Seville. It should be highlighted that the use of these technologies is easier to implement in stabled systems due to their greater homogeneity in animal management. These results are evident in the farms studied that have used these tools (mainly IS and ISC).

Technical, professional advice and training in the management of animal feed are key elements for the reduction of GHG emission levels in goat farming. Strategies based on optimizing the use of inputs through correct rationing, eliminating the excessive and unnecessary use of concentrates, together with the creation of batches providing differentiated diets adapted to the productive level of animals, are necessary for the different production models studied, particularly for stabled systems (IS and ISC). The application of these measures leads to the reduction in the amount of nitrogen (N) and phosphorus (F), thus adjusting the contribution of crude protein, improving digestibility, and reducing the impact of livestock due to a decrease in N and total P excreted and in total NH_4_ emissions [39]. Another main element is the adequate choice of the origin of the raw materials used for animal feeding. The use of local agricultural production, through agreements with cooperatives and producers in the territory and the use of by-products from the area (e.g., bran, molasses, oilseed cakes, olive grove by-products, etc.) contribute to the circular economy without reducing the production and quality of the milk obtained [40].

Particularly, in the case of stabled production systems (IS and ISC), stored manure is an important source of GHG emissions. Although the management and impact of slurry are conditioned by the application of current legislation and, thus, farm facilities (tanks, pits, or storage ponds), a correct subsequent use can contribute to reducing GHG emissions into the atmosphere. Traditionally, slurry has been used as an organic amendment in crops [41], both one’s own and from local farmers, although the increase in its production and the progressive restrictions on its use have accelerated its participation in alternative processes such as anaerobic digestion, which can significantly decrease the emission, combustion and/or storage of methane on the farm [42]. The use of small anaerobic digesters at the farm level that take advantage of the slurry generated can contribute to its energy independence, since biogas can be used as a renewable energy source instead of the use of fossil fuels [43]. This option could be complementary to the use of vegetable biomass (i.e., olive husk) or to the installation of photovoltaic energy on farms, thus reducing emissions linked to fossil fuels [27].

In the case of pastoral systems (GS and PS), actions contributing to the increase in availability and quality of natural and cultivated grasslands are recommended, such as (i) adopting controlled grazing techniques [44], allowing the optimization of pasture production, and controlling spatial and temporal mobility of livestock with new technologies (e.g., tracking collars); (ii) improving the recycling and cycling of nutrients in crops; and (iii) introducing quality autochthonous herbaceous species (e.g., legumes) that contribute to balancing the forage supply. These actions contribute to pastoral systems reaching maximum food autonomy, reducing dependence on external food that has higher environmental costs due to their production and transportation system. Likewise, they would contribute to reducing the GHG emitted into the atmosphere by increasing the carbon sink ability of the ecosystems grazed, giving rise to low-carbon livestock production [37]. In this sense, the lack of consensus on a reference method and data to explain changes in carbon sinks and stocks in vegetation and soil remains a major barrier to their evaluation. New tools, methodologies, and protocols must be developed to assess the contribution of pastoral livestock in the regulation of ecosystem services and compute them when allocating GHG emissions for the calculation of the CF.

Despite the widespread concern for reducing GHG emissions to mitigate climate change, the contribution of each production system to environmental sustainability cannot be measured solely in terms of the value of the CF. In this sense, Knietzko [45] indicates the need to move beyond the carbon tunnel vision with a sustainability data strategy. There are many other elements that determine this contribution and place grazing systems on a different level, which must be supported and protected, given the fragility of these systems. Designing compensation regimes for the ecosystem services provided by pastoral systems could be a tool to improve the competitiveness of these systems [46], which are otherwise at a disadvantage compared to permanently establishing intensive systems, both from an economic and productive point of view [47]. Areas with low cropping potential (e.g., arid or mountainous areas), such as the farms studied, depend on grazing [14,17]. While vegetation plays a critical role in mitigating climate change, it also plays an important role in supporting global biodiversity and providing many goods and services to humans [9,48]. Grazing livestock contributes not only to the production of high-quality livestock products linked to the territory (provisioning services) but also to the prevention of forest fires, the improvement of biodiversity, the improvement of pastures through organic fertilization, the dispersal of seeds, the maintenance of rural populations, etc. (regulation and cultural services) [48]. In these areas, therefore, the abandonment of grazing can have serious consequences.

## 4. Conclusions

The obtained results bring to light the moderate carbon footprint values associated with the production of goat’s milk from Andalusian autochthonous breeds, compared to other studies, with no differences among the production models analyzed. The lack of consensus on the use of specific milk standardization equations is an obstacle to carrying out comparisons on the impact of goat farming in terms of greenhouse gas emissions. Additionally, the lack of consensus regarding a reference method and a suitable database to explain changes in carbon sink and storage through vegetation and soil remains a major barrier to correctly estimating the impact of land-based livestock systems. Even more, including sink values in the carbon footprint calculation is a relevant aspect that allows an adequate assessment and a more realistic view of the environmental impact of these systems.

The need for dairy goat farming to be environmentally sustainable turns out to be an undeniable fact in the present global background, and one of the keys, although not the only one, is minimizing greenhouse gas emissions at the farm level. In this sense, the use of a standardized carbon footprint assessment results in a relevant tool for quantifying these emissions and for, particularly, identifying and understanding the elements of livestock management that contribute to their reduction. In this regard, the use of management tools at the farm level (such as AMALTEUS) allows the joint generation of technical, economic, and environmental indicators for decision making, resulting in a really useful approach.

Finally, the genetic improvement and the efficiency of reproductive and feeding management, including grazing, the optimal use of farm resources, proper management of manure, or the use of local products for animal feed, depending on the production model in question, contribute to moving towards a low-carbon goat production.

## Figures and Tables

**Table 1:** Inputs and outputs for each dairy goat production system monitored in 2018. IS: indoor systems without associated crops (*n* = 8); ISC: indoor systems with associated crops (*n* = 3); GS: grazing systems with high feed supply (*n* = 5); and PS: pastoral systems (*n* = 5). In the same row, different letters indicate significant differences (*p* ≤ 0.05). Mean ± S.E.

	IS	ISC	GS	PS	*F*	*p*-Values
**Inputs**						
Concentrate supply (kg goat^−1^ year^−1^)	499 ± 26 ^a^	465 ± 19 ^a^	568 ± 9 ^a^	258 ± 20 ^b^	74.86	0.003
Fodder supply (kg goat^−1^ year^−1^)	490 ± 32 ^a^	342 ± 88 ^ab^	191 ± 1 ^bc^	41 ± 25 ^c^	32.54	0.001
Fuel (L goat^−1^ year^−1^)	6 ± 3	3 ± 1	6 ± 2	2 ± 1	0.61	0.617
Electricity (kwH goat^−1^ year^−1^)	47 ± 12	27 ± 2	42 ± 13	13 ± 7	1.72	0.200
Mineral fertilizer (kg ha^−1^ year^−1^)	--	110 ± 56	56 ± 34	57 ± 35	0.54	0.663
**Outputs**						
Milk sold (kg goat ^−1^ year^−1^)	578 ± 21 ^a^	489 ± 37 ^a^	508 ± 37 ^a^	320 ± 23 ^b^	17.05	0.003
Meat sold (kg goat ^−1^ year^−1^)	6 ± 1	4 ± 3	5 ± 1	6 ± 1	0.50	0.686

**Table 2:** Total emissions, carbon sequestration by vegetation, and carbon footprint values (kg CO_2_-eq kg^−1^ FPCM) obtained for each farming system studied. The results are presented according to the standardization equation used to calculate the functional unit (kg fat and protein-corrected milk). Equation (1): proposed by Mancilla-Leytón et al. [7]; Equation (2): proposed by Robertson et al. [25]; and Equation (3): proposed by FAO [24]. IS: Indoor systems without associated crops (*n* = 8); ISC: indoor systems with associated crops (*n* = 3); GS: grazing systems with high feed supply (*n* = 5); and PS: pastoral systems (*n* = 5). In the same row, different letters indicate significant differences (*p* ≤ 0.05). Mean ± S.E.

	IS	ISC	GS	PS	*F*	*p*-Values
**Total Emission**						
Equation (1)	1.42 ± 0.05	1.43 ± 0.14	1.58 ± 0.25	1.61 ± 0.27	1.19	0.34
Equation (2)	1.04 ± 0.03	1.04 ±0.11	1.15 ± 0.18	1.17 ± 0.20	1.24	0.33
Equation (3)	1.20 ± 0.04	1.21 ± 0.12	1.33 ± 0.08	1.36 ± 0.17	1.38	0.37
**Carbon Sequestration**						
Equation (1)	--	0.09 ± 0.06 ^c^	0.27 ± 0.09 ^b^	0.66 ± 0.14 ^a^	7.38	0.01
Equation (2)	--	0.07 ± 0.04 ^c^	0.20 ± 0.07 ^b^	0.48 ± 0.10 ^a^	7.39	0.01
Equation (3)	--	0.08 ± 0.05 ^c^	0.23 ± 0.08 ^b^	0.58 ± 0.12 ^a^	7.42	0.01
**Carbon footprint** **(emission-sequestration)**						
Equation (1)	1.42 ± 0.05	1.34 ± 0.09	1.31 ± 0.23	0.95 ± 0.22	3.02	0.58
Equation (2)	1.04 ± 0.03	0.97 ±0.07	0.95 ± 0.17	0.69 ± 0.16	2.95	0.63
Equation (3)	1.20 ± 0.04	1.13 ± 0.10	1.08 ± 0.17	0.78 ± 0.18	3.01	0.57

## Data Availability

Data is contained within the article.
